# An Overview of Autophagy in Hematopoietic Stem Cell Transplantation

**DOI:** 10.3389/fbioe.2022.849768

**Published:** 2022-05-23

**Authors:** Soheila Montazersaheb, Ali Ehsani, Ezzatollah Fathi, Raheleh Farahzadi, Ilja Vietor

**Affiliations:** ^1^ Molecular Medicine Research Center, Tabriz University of Medical Sciences, Tabriz, Iran; ^2^ Student Research Committee, Tabriz University of Medical Sciences, Tabriz, Iran; ^3^ Department of Clinical Sciences, Faculty of Veterinary Medicine, University of Tabriz, Tabriz, Iran; ^4^ Hematology and Oncology Research Center, Tabriz University of Medical Sciences, Tabriz, Iran; ^5^ Institute of Cell Biology, Medical University of Innsbruck, Biocenter, Innsbruck, Austria

**Keywords:** autophagy, hematopoietic stem cells, hematopoietic stem cells transplantation, cellular mechanism, cell therapy

## Abstract

Autophagy is a fundamental homeostatic process crucial for cellular adaptation in response to metabolic stress. Autophagy exerts its effect through degrading intracellular components and recycling them to produce macromolecular precursors and energy. This physiological process contributes to cellular development, maintenance of cellular/tissue homeostasis, immune system regulation, and human disease. Allogeneic hematopoietic stem cell transplantation (HSCT) is the only preferred therapy for most bone marrow-derived cancers. Unfortunately, HSCT can result in several serious and sometimes untreatable conditions due to graft-versus-host disease (GVHD), graft failure, and infection. These are the major cause of morbidity and mortality in patients receiving the transplant. During the last decade, autophagy has gained a considerable understanding of its role in various diseases and cellular processes. In light of recent research, it has been confirmed that autophagy plays a crucial role in the survival and function of hematopoietic stem cells (HSCs), T-cell differentiation, antigen presentation, and responsiveness to cytokine stimulation. Despite the importance of these events to HSCT, the role of autophagy in HSCT as a whole remains relatively ambiguous. As a result of the growing use of autophagy-modulating agents in the clinic, it is imperative to understand how autophagy functions in allogeneic HSCT. The purpose of this literature review is to elucidate the established and implicated roles of autophagy in HSCT, identifying this pathway as a potential therapeutic target for improving transplant outcomes.

## Introduction

Autophagy is a fundamental catabolic process for maintaining cell homeostasis through recycling intracellular components and regulating metabolic functions. As a lysosome-dependent degradation process, autophagy facilitates the degradation of intracellular materials such as damaged organelles, misfolded proteins, and lipids by delivering to lysosomes and subsequent recycling them (Galluzzi et al., 2017). Autophagy is an evolutionarily conserved process from yeast to mammals that provide energy to the cells when they are deprived of nutrients or under metabolic stress (Vijayakumar and Cho, 2019).

During hemostasis, all organisms and cells have a constitutive level of autophagy by which cytoplasmic content is turned over. Multiple cellular stresses can induce the autophagy pathway, including nutritional deficiencies, DNA damage, oxidative stress, endoplasmic reticulum stress, hypoxic status, infection, and other stresses (Ravanan et al., 2017). The autophagy term was first described by Christian De Duve in 1963. As the name implies, autophagy (self-eating) is the process of sequestering intracellular cargo via a network of interconnected double-membraned vacuoles, called autophagosomes (Klionsky, 2008; Mizushima, 2018).

**FIGURE 1:**
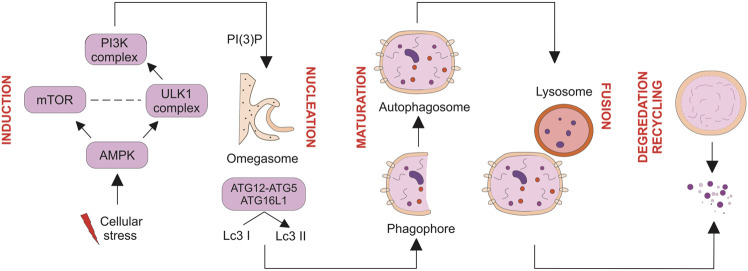
Multiple steps of autophagy. Autophagy is negatively and positively regulated by mTOR and AMPK, respectively. Maturation and elongation of the autophagosome membrane involve two ubiquitin-like conjugation steps, including the conjugation of ATG12 to ATG5 and the conversion of LC3 I to LC3 II. Fully formed autophagosomes fuse with lysosomes to degrade their intracellular content and recycle macromolecule components. Nucleation of the autophagosome structure involves generating PI (3) P at the omegasome.

Based on the sequestration mechanism, three types of autophagy were identified: microautophagy, macroautophagy, and chaperone-mediated autophagy, in which macroautophagy is a widely explored form of autophagy. The present review focuses primarily on macroautophagy, which is hereafter called autophagy. Autophagosome is the main hallmark of autophagy. Autophagosomes contain cytoplasmic material such as cytoplasm, organelles, and proteins, engulfed by double membranes (Hollenstein and Kraft, 2020). During autophagy, several sequential events occur inside cells which start with induction, phagophore nucleation, elongation/maturation of the autophagosome membrane, the fusion of the autophagosome with the lysosome, and finally, degradation and recycling of nutrients by lysosomes’ protease (Figure 1) (Devkota, 2017; Dikic and Elazar, 2018; Chang, 2020).

The initiation and execution of autophagy rely on the activity of two ubiquitin-like conjugation systems and the function of multi-protein complexes and pathways. In light of the similarity of autophagy in the yeast and mammalian cells, it has been reported that a conserved subset of autophagy-related genes (Atg) and proteins contribute to the autophagy process (Nishimura and Tooze, 2020). In an initial study using *Saccharomyces cerevisiae*, it was revealed 18 Atg proteins are required for autophagosome formation, which classified into six functional groups, including Atg8/Atg12 ubiquitin-like conjugation systems, the class III phosphoinositide 3-kinase complex I (PI3KC3-C1), the Atg2–Atg18 complex, Atg9 transmembrane protein, and the Atg1 kinase, which is all conserved from yeast to mammals, with a few exceptions. Among Atg groups, the Atg1 kinase activity is regarded as the major upstream factor (Mizushima et al., 2011; Noda and Fujioka, 2015). More than 30 Atg genes are involved in the autophagic process.

Autophagy induction is triggered by various intracellular and extracellular stimuli (Mazure and Pouysségur, 2010). Autophagy is mediated by activation of unc-51-like kinase consisting of ULK1/2, followed by PI3KC3-C1 activation. In response to stimulating factors, the ATG13 anchors ULK1 to form a pre-autophagosomal structure (PAS), and then all Atg proteins gather onto the PAS, an essential site for autophagosome formation (Yamamoto et al., 2016). PAS plays a critical role during the induction of autophagy (Kotani et al., 2018). The mammalian ULK1 complex (homologous to Atg1 in yeast) consists of ULK1, ATG13, FIP200, and ATG101 that act as initiation complex in autophagy. ATG13 is an essential protein for the interaction of ULK1 with FIP200 (Atg11 and Atg17 in yeast) and acts as a scaffold for the assembly of Atg protein in the downstream path. Once PAS is targeted by ATG13 and ULK1, all ATG proteins are involved and localized into the PAS to initiate autophagy (Kawamata et al., 2008).

The mature autophagosome is generated by nucleation of the several Atg proteins at PAS structure, followed by membrane isolation that leads to autophagosome maturation. Nucleation process is mediated by ULK1/Atg1 complex by forming a complex of the ULK1/Atg1 protein with Atg13, FIP200/Atg17, Atg29, and Atg31. Then this complex can generate PAS scaffold complex, and PI3K is gathered to the PAS and formed phagophore. Afterward, the other functional ATG9A system, ATG12-conjugation system, and light chain 3-phosphatidyl ethanolamine (LC3-PE) conjugation system are targeted to the PAS and involved in assembly and formation of autophagosome (Lamb et al., 2013).

This double membrane structure encapsulates cytoplasmic materials as it matures. In this path, two ubiquitin-like conjugation systems, including Atg12-Atg5-Atg16L1 and microtubule-associated LC3-PE, control elongation and closure of the autophagosome. Subsequently, the fusion of the autophagosome with lysosome can form an autolysosome which facilitates the degradation of luminal contents (He and Klionsky, 2009; Li et al., 2020). The degraded contents are recycled for synthesizing new proteins and energy production (Marshall and Vierstra, 2018). Collectively, autophagy is a well-recognized pathway for maintaining cellular homeostasis.

As described above, autophagy exhibits a cytoprotective effect in response to various stimuli, such as nutrient deprivation, cytokine-induced stress, reactive oxygen species (ROS), hypoxic conditions, and endoplasmic reticulum (ER) accumulation. Apart from the abilities mentioned, autophagy can also be implicated in multiple physiological processes, such as cellular differentiation, development, and remodeling (Wang et al., 2019). In addition, autophagy plays a critical role in innate and adaptive immunity through eradicating pathogens, presenting antigens, and controlling inflammation. Despite its protective activity, excessive autophagy can also result in cellular death (Maiuri et al., 2007). An autophagic response can be either cell survival or death, depending on the cellular context and stimuli. Relying on this intricate nature, autophagy plays a fundamental physiological role in various cellular events (Cao et al., 2021).

### The Physiological Importance of Autophagy

Under normal physiologic conditions, autophagy is a process that occurs at a basal, constitutive level in virtually all eukaryotic cells. Autophagy-deficient cells exhibit abnormal protein aggregation and mitochondrial dysfunction (Yin et al., 2016). In keeping with this concept, autophagy contributes to maintaining cellular homeostasis through clearance of protein aggregates and damaged organelles, which are normally resistant to degradation via the ubiquitin-proteasome system (UPS) under normal growth conditions (Wang and Le, 2019).

**FIGURE 2:**
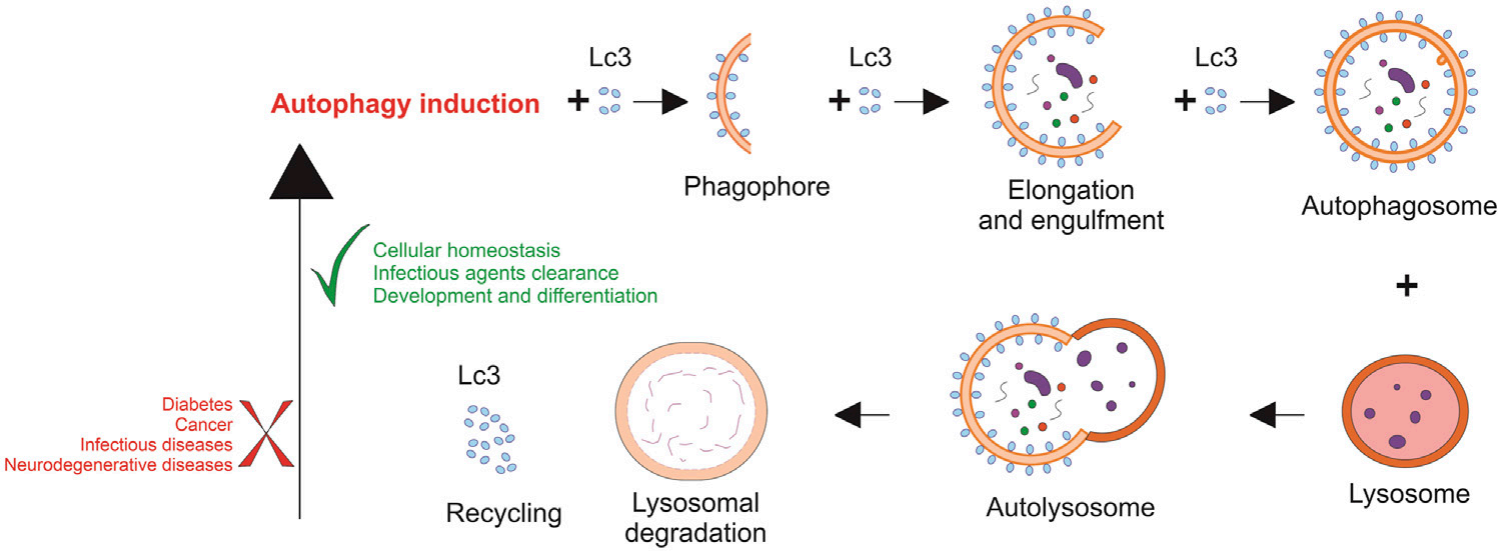
Dysregulated autophagy is involved in several human diseases. Autophagy is critical in a wide range of normal human physiological processes and contributes to maintaining cellular homeostasis. Therefore, many diseases are associated with the misregulation of the autophagic process.

Aside from maintaining cellular homeostasis, autophagy drives the rapid cellular changes necessary for proper mammalian development and differentiation. ATG gene knockout mice showed severe impairments during differentiation and developmental process in mammals (Hu et al., 2019). Furthermore, erythrocyte maturation is dependent on the autophagic degradation of mitochondria in later stages (Grosso et al., 2017). It has also been revealed that autophagy plays a pivotal role in white adipogenesis by expanding white adipose tissue (Figure 2) (Jing and Lim, 2012; Clemente-Postigo et al., 2020).

Autophagy also plays a substantial role in preventing viral infection by removing the virus and regulating the immune system to promote virus clearance (Xiao and Cai, 2020). In the case of bacterial infection, autophagy can stimulate macrophages’ activity and provide protective effects against bacterial toxins. Autophagy also can enhance bacterial degradation by delivering antimicrobial peptides to the lysosomes (Cemma and Brumell, 2012). Besides, host cells control the evasion of *Mycobacterium tuberculosis* by upregulation of autophagy. This elaborate cellular mechanism targets bacteria and eliminates or decreases the bacterial loads within the infected cells (Bento et al., 2015). Several growing research has been conducted to determine whether autophagy is involved in combating COVID-19. In this context, Shang et al. (2021) showed that autophagy-inhibiting factors might have beneficial effects against COVID-19 (Shang et al., 2021). Together, autophagy can contribute to human disease through innate and adaptive immunity (Mizushima and Levine, 2020).

### Hematopoietic Stem Cells

Hematopoiesis is a continuous and tightly regulated process by which adult hematopoietic stem cells (HSCs) supply both myeloid and lymphoid lineages. Hematopoiesis is a prerequisite long-life phenomenon for producing short-lived blood cells with a constant turnover. In other words, hematopoiesis is a well-regulated biological process that provides a balance between HSCs quiescence, self-renewal, activation as well as proliferation, and differentiation (Fathi et al., 2021). Perturbations of the hematopoietic system can develop blood disorders such as anemia (under-production of red blood cells) and leukemia (abnormal proliferation and aberrant maturation of leukocytes) (Sawai et al., 2016; MacLean et al., 2017).

Several studies have shown that mice lacking functional autophagy genes, including FIP200, Atg7, and Atg12, experienced a severe loss of HSCs. These data indicate the importance of these genes in the function, integrity, and maintenance of the HSCs pool, especially long-term HSCs, and mature progenitor cells (Liu et al., 2010; Mortensen et al., 2011; Gomez-Puerto et al., 2016; Rodolfo et al., 2016). Indeed, in the absence of Atg7 or FIP200 genes, HSCs failed to restore lethally irradiated syngeneic recipients, thereby highlighting a crucial role of autophagy in HSC self-renewal. As confirmed by *in vitro* assessment using the serial colony-forming unit, Atg7-deficient HSCs displayed a significant reduction in colony formation (Mortensen et al., 2011). Furthermore, Atg7-deficient HSCs cells exhibited damaged mitochondria accumulation with increased ROS and DNA damage (Mortensen et al., 2011; Ianniciello and Helgason, 2022).

According to studies, quiescent HSCs are low in oxidative phosphorylation, but after activation, they switch toward a high level of oxidative phosphorylation (Papa et al., 2019).

Atg12-deficient HSCs showed increased mitochondrial content, activated metabolic activity, and increased myeloid differentiation, similar to the aging-associated phenotype (Ho et al., 2017). As evident by transplantation experiments, Atg12-deficient HSCs failed to regenerate and self-renew properly.

Relying on these findings, autophagy has a peculiar role in maintaining HSCs quiescence and self-renewal capacity by removing activated mitochondria as a mechanism for controlling oxidative metabolism. Autophagy-mediated metabolic activities are associated with epigenetic reprogramming, as evidenced by alterations in DNA methylation patterns of Atg12-deficient HSCs. Accordingly, basal autophagy determines HSCs fate through epigenetic regulation of mitochondrial content (Ho et al., 2017).

Due to the importance of autophagy in stem cell maintenance, HSCs can also induce autophagy in response to metabolic stresses to protect against cell death. In response to autophagy induction, a high level of transcription factor FOXOA3 is expressed in HSCs (Warr et al., 2013), which targets and promotes the transcription of pro-autophagy genes (Fitzwalter and Thorburn, 2018). Consequently, by maintaining persistent levels of FOXOA3, HSCs can rapidly induce autophagy-related genes when faced with metabolic stress (Warr et al., 2013). Consistent with these observations, Foxo3a-null aged mice showed a reduction in HSCs reservoir, with decreased self-renewal capacity and defects in stem cell quiescence (Miyamoto et al., 2007). In the case of aging, aged HSCs exhibited greater basal autophagy (around 30%) compared with their younger counterparts (Revuelta and Matheu, 2017a). It has been revealed that enhanced autophagy in aged HSCs was linked to the lifelong survival and maintenance of old HSCs in the aging bone marrow niche with nutrient deficiency (Frans, 2019).

It is crucial to understand why aged HSCs exhibit heterogeneous features of autophagy and investigate the reasons for enhanced autophagic activity, as opposed to diminished autophagy of other old tissues (Revuelta and Matheu, 2017b).

It has been found that aberrant autophagy in HSCs develops hematological disorders, such as anemia. According to Mortensen et al. (2011), mice knocked out for Atg7 gene caused anemia and death between 8 and 14 weeks. These results indicated that loss of autophagy leads to defective mitochondria clearance and severe anemia *in vivo* (Mortensen et al., 2010).

Autophagy is a fundamental event in erythrocytic differentiation since it clears cytoplasmic organelles and removes nuclei. Autophagosome formation needs the ULK1-Atg13-FIP200 structure, which is inhibited by PI3K/AKT/mTOR activation. According to the result by Orsini, the TNFα/neutral sphingomyelinase/ceramide pathway inhibited autophagy in erythropoietin-induced CD34/HSPCs. In fact, TNFα and ceramide could phosphorylate mammalian target of rapamycin (mTOR) S2448 and ULK1S758 and inhibit Atg13S355 phosphorylation, that eventually blocked autophagosome formation (Orsini et al., 2019). Noteworthy, loss of autophagy did not equally affect all hematopoietic lineages, implying its diverse impact on hematopoietic differentiation (Rožman et al., 2015; Simon et al., 2019).

### The Cellular Mechanisms Involved in Autophagy During Hematopoietic Differentiation

As described in the previous session, several signaling hubs induce autophagy, including mTOR and about 50 adenosine monophosphate-activated protein kinase (AMPK) that converge on the ULK1 complex to trigger autophagosome nucleation. This is mediated by targeting the PI3KC3, containing vacuolar protein sorting 34 (VPS34) and BECLIN 1. Maturation of the autophagosome is mediated by the local lipidation through conjugating phosphatidylethanolamine to LC3 protein (LC3- I). Afterward, the lipidated LC3 (LC3-II) is directed to autophagosome membranes for the elongation process. Mammalian cells contain multiple homologs of Atg8, some of which recruit selective autophagy receptors through LC3-interacting regions (LIR domains) (Birgisdottir et al., 2013; Slobodkin et al., 2013).

Selective autophagy is regarded as a vital mechanism for cell-autonomous immunity. Three autophagy receptors are SQSTM1/p62, NDP52, and Optineurin. In this context, cytoplasmic components are selected and tagged with ubiquitin for recognition by autophagy receptors (Gubas and Dikic, 2022).

Although selective autophagy and basal autophagy intersect with the Atg8 family, their dynamic interplay remains unclear. Indeed, molecular autophagy is a pleiotropic phenomenon, and its signaling cascades interact with other pathways such as ubiquitinylation events, cellular death, phagocytic activity, cell cycle machinery, and secretion. There are many details and open questions regarding autophagic pathways and their cross-talk (Lindqvist et al., 2015); these details will not be addressed here; meanwhile, they may help settle some controversies concerning autophagy in hematopoietic systems. Despite initial descriptions of autophagy as a bulk degradative and starvation-induced activity, increasing evidence suggests that a complex network mediates autophagy degradation such as selection, autophagic activity, and timing of autophagy-dependent degradation (Stolz et al., 2014).

Specific cargo selection is mediated by several factors, including degradation cues (e.g., the ubiquitin-code) (Shaid et al., 2013; Vainshtein and Grumati, 2020), selective autophagy receptors (Farré and Subramani, 2016; Kirkin and Rogov, 2019), and the lysosome-directed process with high selectivity (e.g., chaperone-mediated autophagy, microautophagy, and LC3-associated phagocytosis) (Li et al., 2012; Martinez et al., 2015; Yang et al., 2019). Recent discoveries have revealed a wide variety of selective autophagy cargo with specific receptors for misfolded or aggregation-prone proteins (aggrephagy), dysfunctional mitochondria (mitophagy), peroxisome as dynamic organelle (pexophagy), surplus ER (reticulophagy), nucleus components (nucleophagy), and subunits of ribosomes (ribophagy) (Mancias and Kimmelman, 2016; Gatica et al., 2018).

In selective autophagy, damaged organelles or protein aggregates are removed through substrate ubiquitination and autophagy receptors. SQSTM1/p62, NDP52, and Optineurin can specifically bridge the ubiquitinated components with the inner membrane of the autophagosome (Lamark and Johansen, 2021). Nguyen et al. demonstrated that p62 knock-down could impair the expansion and colony-forming capacity of the oncogene-transformed cells *in vitro*, implying the prominent role of p62 in leukemia development (Shaid and Brandts, 2017). Another study by Li et al. showed that XRK3F2, as a new P62 inhibitor exerts its antileukemic activity through impairing mitophagy in leukemia-initiating cells. This led to a reduced colocalization between LC3 and mitochondrial proteins accompanied by an accumulation of dysfunctional mitochondria (Li et al., 2021).

Despite extensive research on the autophagic molecular networks (Atgs and related upstream signaling pathways) over the last 2 decades, little is known about its interaction with cell biology in determining cell fate. Noteworthy, numerous cellular systems have been affected by autophagy regulation, including cell differentiation, self-renewal, survival, and cell death (Mizushima and Levine, 2010; Wang et al., 2019).

In an *in vivo* setting, defective differentiation in the absence of autophagy is well defined in multiple hematopoietic cell types; however, most of these studies only address the effects of cargo degradation on cell fate in a site-specific manner. The lack of knowledge regarding cross-tack of autophagy and associated differentiation events may be improved by deleting only one type of selective autophagy factor without affecting the LC3- conjugation machinery (e.g., specific deletion BNIP3L-mediated mitophagy).

Besides, this can be accomplished by purifying and observing the dynamic alterations of autophagosomal components during differentiation. These observations improve our understanding of how cell differentiation is affected by transcription- and growth factor-independent mechanisms (Riffelmacher and Simon, 2017).

### Hematopoietic Stem Cell Transplantation

HSCT is the most effective treatment option for most bone marrow-derived diseases, oncologic malignancies, metabolic disorders, and immune deficiency (Zimowski and Chandrakasan, 2018; Fathi et al., 2019). Indeed, HSCT is applied after chemotherapy or radiation therapy to consolidate remission and provide a durable treatment in severe hematologic malignancies (Fathi et al., 2020). HSCT can be categorized into two main types of procedures as follows: autologous HSCT (patients’ stem cells are collected) and allogeneic HSCT (stem cells come from a separate individual who donates HSCs) (Fraint et al., 2021). Autologous transplantation has the advantage of eliminating long-term immunosuppressive therapy, on the other hand not producing a graft-versus-leukemia (GVL) effect. Interestingly, the GVL effect of donor cells is essential to the curative potential of HSCT that can be mediated by donor‐derived immunity.

Allogeneic HSCT treats various hematological malignancies such as relapsed or high-risk leukemia. Importantly, this type of transplant can provide GVL effects to eradicate leukemia (Singh and McGuirk, 2016). Allogeneic HSCT can restore the aberrant auto-reactive immune responses with a healthy immunity to be tolerated with allo- and auto-antigens. Despite its superior advantages, the application of allogeneic HSCT is constrained due to inherent toxicities, including graft failure and graft-versus-host disease (GVHD) (Ros-Soto et al., 2021).

Graft failure or graft rejection is a serious and life-threatening complication of allogeneic HSCT.

Generally, graft failure can be categorized as primary or secondary. Primary graft failure can occur due to the lack of initial donor cells engraftment (less than 95%). In contrast, secondary graft failure is defined by the loss of donor cells following initial engraftment (Ozdemir et al., 2018).

GVHD occurs when donor T cells recognize the membrane antigen of the recipient as foreign and respond against allogeneic antigen-bearing cells. It is important to note that GVHD can be acute or chronic, and both types can lead to significant death and morbidity after transplantation (Dickinson et al., 2017). Acute GVHD appears within the first days after transplantation and activates innate immune receptors mediated by T-helper type 1 (Th1) responses, leading to cytokine storms. This phenomenon occurs in about 40% of transplants, which eventually induce apoptosis in the gastrointestinal tract, liver, and skin. GVHD is induced when antigen-presenting cells (APCs) present host alloantigen to naive donor T cells (Zeiser and Blazar, 2017).

**FIGURE 3:**
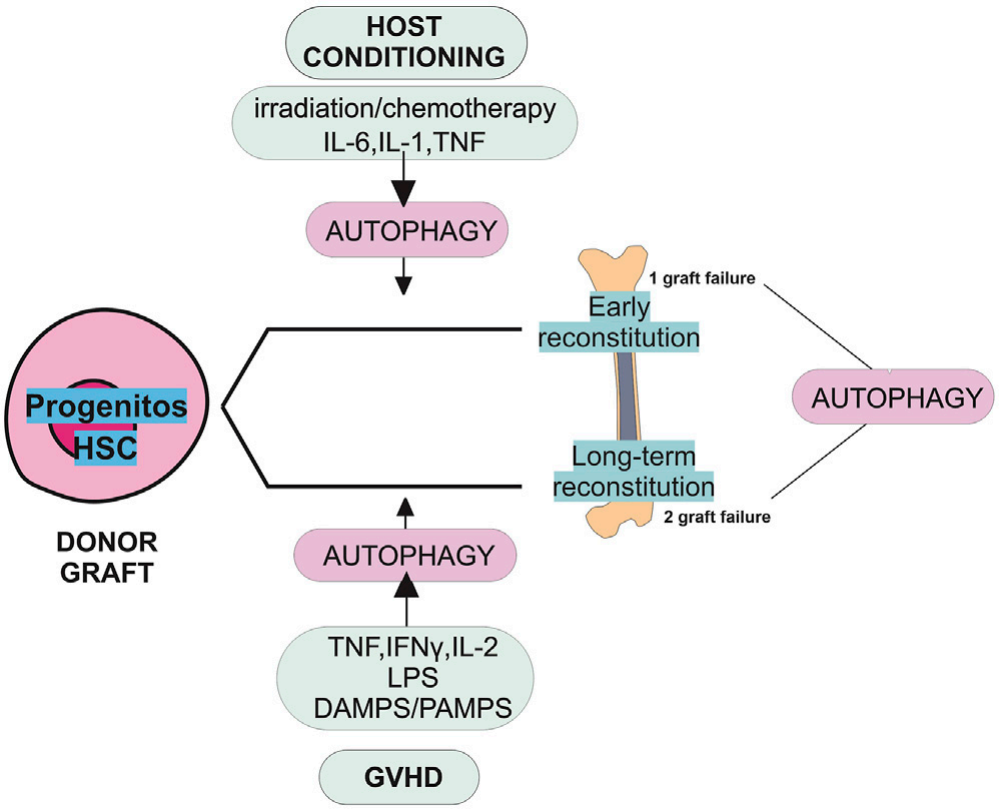
Role of autophagy in graft failure. The schematic view in this diagram shows the potential roles of autophagy in primary and secondary graft failure following allogeneic HSCT. Graft failure may be manifested as primary (lack of initial engraftment of donor cells) or secondary (loss of donor cells after initial engraftment). The absence of autophagy may promote the risk of primary and secondary graft failure. The stress induced by preconditioning regimens such as irradiation, chemotherapy, and cytokines could induce autophagy. Following autophagy induction, pharmacological reagents could promote progenitor cell differentiation and early reconstitution improvement. The induction of autophagy may help HSCs overcome the stress situation caused by GVHD and allow their long-term reconstitution. Autophagy is necessary for the survival and repopulating function of HSCs. HSCs participate in the long-term reconstitution following HSCT. In a stress situation induced by GVHD, releasing cytokines damage/pathogens-associated molecular patterns (DAMPS/PAMPS) and lipopolysaccharide (LPS) induces autophagy.

Collectively, the curative potential of HSCT depends on the capacity of the transferred HSCs to engraft and restore the patient’s immunity that had been weakened by chemotherapeutic or irradiation therapy. Furthermore, the GVL effect is essential for allograft stem cells to clear hematological malignancies. Although important insights have been revealed into the role of HSC in repopulating function, differentiation, immune cell activation, and inflammation, little is known about the role of autophagy in these processes. The success of HSCT depends on the reconstitution of hematopoiesis and immune reaction. The hematopoiesis is tightly regulated by various factors, including intrinsic and extrinsic signals and a subpopulation of HSCs known as long-term HSCs. These cells are characterized by their longevity, quiescence state, self-renewal, and differentiation ability (Figure 3) (Leveque et al., 2015; Mayani, 2016; MacLean et al., 2017).

Interestingly, under clinical stress, particularly in HSCT and mobilization after chemotherapy, HSCs can rapidly produce new mature blood cells. Therefore, HSC requires more autophagy activity than other cells to preserve their unique features and to survive long-term survival (Nguyen-McCarty and Klein, 2017).

Due to the inhibitory effect of Bafilomycin A1 on autophagosome-lysosome fusion and possible decrease in self-renewal capacity, HSC was treated with Bafilomycin A1 before transplantation (Mauvezin and Neufeld, 2015). In the presence of Bafilomycin A1; HSC derived from aged mice showed reduced self-renewal capability than young mice, as detected by colony-forming unit assays. Based on this, HSCs require a high level of autophagy to survive during the aging process (Warr et al., 2013). Additionally, maintaining autophagy in HSCs during aging may reduce the risk of blood diseases characterized by stem cell failure such as myelodysplasia. After recovery of the hematopoietic system, HSCs re-enter the quiescence state to sustain long-term self-renewal. In this context, mice lacking Atg7 failed to produce long-term HSCs; however, a simultaneous expansion of HSCs occurred in the bone marrow, highlighting the loss of quiescent HSCs. Furthermore, HSC expansion induced mitochondrial mass accumulation and ROS production accompanied by DNA damage, proliferation, and apoptosis (Mortensen and Simon, 2010).

As defined in the previous part of this review, mitophagy drives mitochondria removal through autophagy, critical for HSC maintenance and preserving quiescence (Joshi and Kundu, 2013). Overall, the results show that autophagy is a fundamental process for the long-term survival and function of HSC.

Additionally, HSC grafts enriched with more mature committed progenitor cells to facilitate initial engraftment and protection against opportunistic infections, the leading cause of morbidity and mortality following HSCT. It is known that definitive engraftment and expansion of HSCs provide long-term hematopoiesis and immune system reconstruction. Given the well-established role of autophagy in HSCs and progenitor cells, mainly in the syngeneic transplant setting (Warr et al., 2013), modulating autophagy during the early post-transplant phase might influence the quality of engraftment (Leveque et al., 2015).

Before allogeneic HSCT, patients are exposed to a conditioning regimen such as high-dose chemotherapeutic or total body irradiation. This condition, accompanied by T-cell alloreactivity, can create an inflammatory milieu responsible for subsequent complications (Gao et al., 2018). In this complex scenario, HSC survival is highly dependent on autophagy. Interestingly, the mechanism behind secondary graft dysfunction during GVHD remains poorly understood; therefore, it is tempting to hypothesize that autophagy might be required to maintain HSC functionality. Overall, therapeutic strategies aiming to boost the autophagy of HSCs and their progenitors after transplantation may promote their survival and differentiation, thereby improving engraftment (Leveque et al., 2015).

### Autophagy and Tolerance in HSCs

A subset of Tcell called regulatory T cells (Tregs) plays a vital role in immune homeostasis and peripheral tolerance by various immune functions. Several reports in mice and humans have demonstrated the importance of Treg tolerance following HSCT (Hanash and Levy, 2005; Gu et al., 2019; Whangbo et al., 2020). In line with this, clinical studies established that Treg could prevent both acute and chronic GVHD through immunomodulatory effects on the immune system (Edinger et al., 2003; Gu et al., 2019; Whangbo et al., 2020; Guo et al., 2021). In addition, it has been revealed that T reg levels are inversely correlated with disease severity and progression rate (Valverde-Villegas et al., 2015; Romano et al., 2017). In light of these findings, the application of Treg adoptive transfer has promising immunotherapy impacts in preventing acute and chronic GVHD (Michael et al., 2013). Despite this, the limited accessibility of natural Tregs limits its application. To address this issue, natural Treg expansion protocols are being developed to Treg from conventional CD4 T cells (Hippen et al., 2011; Zhang et al., 2013).

Despite the promise of *in vitro* derived Tregs, the stability of FoxP3 and its survival following *in vivo* transfer remain unknown. Indeed, FOXP3 expression is an essential transcriptional factor for the development and activity of T reg. Rapamycin, a potent inhibitor of mTOR known to drive autophagy, is more commonly used as an immunosuppressant in clinical HSCT (Kornblit et al., 2014; Paquette et al., 2018). Natural adoptive transfer studies have demonstrated that rapamycin can suppress conventional T-cell expansion and differentiation while sparing the function of transferred Tregs, a crucial issue in therapeutic strategies (Coquillard et al., 2015). Compared to mice treated with rapamycin, cyclosporine A (CsA) treatment has the opposite impact. Indeed, CsA affects the expansion and development of Treg in pathologic states such as GVHD (Zeiser et al., 2006). Satake et al. (2014) provided evidence that CsA inhibits Treg expansion and inducible Treg production in allogeneic bone marrow transplantation (BMT). In addition, CsA permits IL-2-induced Treg proliferation in the syngeneic setting BMT with IL-2 (Satake et al., 2014), whereas rapamycin completely suppressed IL-2-mediated Treg expansion. In other words, CsA can abrogate the protective effect of IL-2 on allogeneic BMT-induced GVHD, while rapamycin acts inversely. Given the critical role of FoxP3 in HSCT and considering its transient expression following adoptive transfer, co-administration of rapamycin with IL-2 could enhance FoxP3 stability and preserve Treg numbers following allogeneic stem cell transplantation *in vivo* (Satake et al., 2014). Overall, rapamycin is a better option in an allogeneic setting as an adjunct for IL-2 to proliferate Tregs. Besides, rapamycin could alleviate acute GVHD by stabilizing Tregs (Zhang et al., 2013); however, the direct effect of rapamycin on autophagy needs to be elucidated. Based on this evidence, more mechanistic studies are thus required to develop strategies to maximize the survival and stability of Treg in the clinic.

It has been demonstrated that mobilization of HSC with granulocyte colony-stimulating factor (G-CSF) stimulated Treg expansion in both donors and recipient mice after SCT, protecting against GVHD. In this regard, modulatory effects of G-CSF on Treg were analyzed by microarray mRNA expression. Analysis showed overexpression profiles of autophagy-related genes in highly purified Treg from G-CSF–treated mice (MacDonald et al., 2014).

Currently, there is no definitive evidence for autophagy activity in Treg cells. On the other hand, the class III PI3K vacuolar protein sorting (Vps) 34 plays a crucial role in autophagic flux. Mice lacking Vps34 in the T cell lineage exhibited profound autophagy defects, accumulating cellular organelles. In addition, ablation of Vps34 in T cells had a profound impact on T cell function and homeostasis. As a result, aged animals lacking Vps34 developed a wasting syndrome marked by weight loss, inflammation of the intestines, and anemia. This implies that Vps34 is essential for maintaining and functioning Treg cells (Parekh et al., 2013).

It has been revealed that histone deacetylase inhibitors (HDACis) have beneficial effects on inflammation. Emerging evidence has proved that HDACis can suppress immune-mediated complications such as GVHD. In this context, modulating immune responses can be achieved by inhibiting pro-inflammatory cytokines production along with increasing the number and function of Treg cells. Therefore, HDACis can be used as new immunotherapies against GVHD and other immune diseases. (Xu et al., 2021). It has also been revealed that HDAC1 can induce autophagic signals through mTOR inhibition. Accordingly, the combination of rapamycin with HDAC1 is a promising option to upregulate Treg differentiation and expansion, as well as activate autophagic signals. On the whole, the findings indicate a strong link between autophagic flux and Treg maintenance in an *in vivo* setting (Fröhlich, 2017).

### Modulating Autophagy in Clinical Transplantation

Several autophagy-modulating agents have already been approved in clinical transplantation. GVHD is currently controlled by immunosuppressive drugs, many of which affect autophagic pathways. Historically, calcineurin inhibitor is the most common regimen used for GVHD prophylaxis in clinical settings. CsA is typically used to inhibit ongoing immune responses after transplantation by controlling the expansion of T-cells. In addition, CsA can induce autophagy by stimulating endoplasmic reticulum (ER) stress, which eventually leads to phenotypic changes in human tubular and cellular death (Wu et al., 2018). CsA as an immunosuppressive agent is used to reduce transplant rejection rates in many organs. However, CsA’s nephrotoxicity limits its utilization in clinics. Growing evidence indicates that oxidative stress has a vital role in developing side effects. On the other hand, CsA-mediated endoplasmic reticulum stress leads to an increment in mitochondrial reactive oxygen species production, which results in lipid peroxidation and nephrotoxicity. However, recent studies declare that CsA-induced autophagy can alleviate the detrimental impact on ER (Wu et al., 2018). Consistent with these findings, Ciechomska et al. (2013) demonstrated that CsA could induce autophagy in malignant glioma cells by triggering ER stress and mTOR inhibition. Besides, silencing autophagic effectors such as ULK1 and Atg5 contributes to the upregulation of apoptotic proteins in CsA-treated cells (Ciechomska et al., 2013). Like CsA, thiopurine can activate the autophagic pathway, providing protective effects against cytotoxicity (Guijarro et al., 2012). Accordingly, by using immunosuppressants and promoting autophagic activity, it is possible to overcome the intrinsic cytotoxicity of these compounds.

In contrast, chloroquine and hydroxychloroquine inhibit autophagic flux *via* interference with autophagosome-lysosome fusion, mitigating chronic and acute GVHD (Schultz et al., 2002; Khoury et al., 2003). In addition, Bortezomib is reported to be capable of both increasing and decreasing autophagy depending on the type of cell, exhibiting promising results for preventing GVHD in phase II trials (Koreth et al., 2012; Martin, 2018). Relying on these findings, modulating autophagy by pharmacological agents (activator or inhibitor) will likely affect the outcome of a transplant. Noteworthy, blocking autophagy maintains gut homeostasis and prevents Toll-like receptor ligand translocation, reducing activation of APC and donor T cell priming. On the contrary, induction of autophagy after transplantation may reduce the intrinsic cytotoxicity of immunosuppressive agents. Collectively, it is crucial to find out whether the beneficial effects of these agents are at least in part mediated by autophagy (Leveque et al., 2015).

### Modulating Autophagy in Stem Cells

**FIGURE 4:**
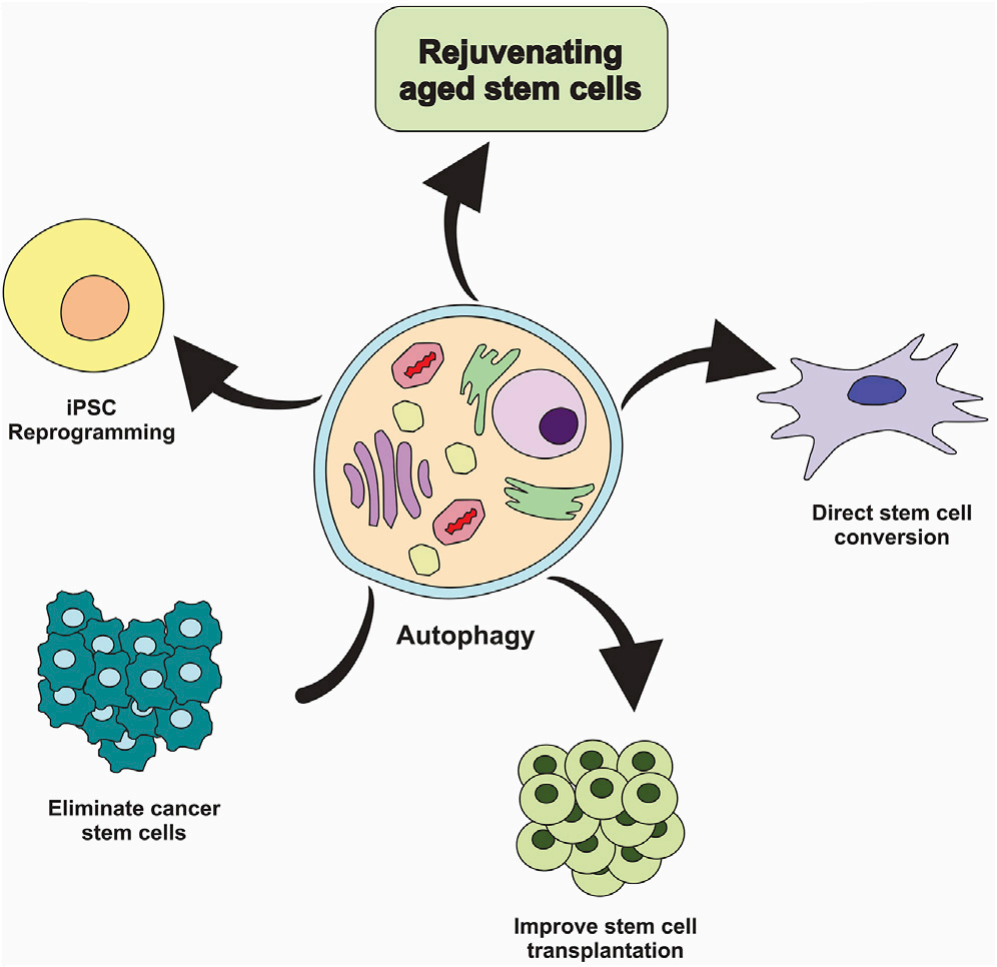
Targeting autophagy in stem cells. Inhibiting autophagy in cancer stem cells could be considered a valid strategy to eliminate tumor-initiating cells and prevent cancer treatment resistance. In contrast, induction of autophagy could improve stem cell transplantation therapies and aged stem cell function, facilitate the conversion of somatic cells into the tissue-specific stem cells and promote reprogramming of somatic cells into the induced pluripotent stem cells (iPSC).

Many studies have revealed the role of autophagy-mediated cell survival in cancers progression; thereby, autophagy inhibition may offer anti-cancer properties. Interestingly, autophagy may have therapeutic benefits in regenerative medicine due to its essential role in preserving normal stem cell function (Figure 4) (Chang, 2020). Several studies show that stimulation of autophagy by genetic and pharmacological means has been linked with increased regenerative capacity and function of aged stem cells. Increased autophagy may delay aging and extend longevity (García-Prat et al., 2016; Leeman et al., 2018). Intriguingly, few studies have explored how autophagy modulation impacts stem cell transplantation.

Stem cell transplantation is an innovative therapeutic modality for regenerating and repairing damaged cells and tissues. This strategy enables stem cells to repopulate the niche and provide long-term correction of degenerative phenotype (Rajabzadeh et al., 2019). However, a variety of limitations impede the stem cell transplantation in the clinic, including difficulties in *ex vivo* expansion of stem cells, a low survival rate of transplanted cells, a decline in the self-renewal of engrafted cells, preferential differentiation, and, more importantly, the risk of emergence of GVHD (Singh and McGuirk, 2016). For instance, HSCs derived from umbilical cord blood have remarkable promise for HSCT. Given clinical use of cord blood as a stem cell source, widespread clinical application of this approach is restricted due to the low yield of hematopoietic stem and progenitor cells within each unit of cord blood.

In a study by Xie et al. (2019), genetic modulation or pharmacological blockade of sphingolipid enzyme could regulate lineage differentiation. Indeed, DEGS1 (delta 4-desaturase, Sphingolipid 1) is needed by HSCs for proper function. In this regard, *ex vivo* treatment of DEGS1 inhibitors such as 4HPR with human cord blood-derived HSCs could enhance the self-renewal capacity of long-term HSCs (Xie et al., 2019). Compared to untreated cells, there was a drastic increase in the repopulation of long-term HSCs using serial transplantation approaches into immune-deficient mice. Improvement of HSCs function *via* 4HPR-mediated activity may be attributed to the activation of proteostasis programs and autophagy along with the unfolded protein response. Thus, identifying compounds that can expand stem cells while preserving their self-renewal capacity holds great promise for the widespread use of stem cell transplantation in the future (Li et al., 2019; Xie et al., 2019).

In addition, various research has revealed that the differentiation of induced pluripotent stem cells (iPSCs) or lineage reprogramming somatic cells into specific lineage can create a massive supply of stem cells. Also, many studies have shown that reprogramming protocols combined with gene-editing technology (CRISPR/Cas9) may prevent transplant-associated complications (Rao et al., 2022). In a model of Huntington’s disease, administration of a small-molecule enhancer of rapamycin 28 (SMER28) can modestly increase autophagy and subsequent clearance of autophagy substrates (Whitmarsh-Everiss and Laraia, 2021). These small molecules exhibit efficacy in reprogramming fibroblast cells into neural stem cells (NSCs). In this context, Zhang et al. (2013) showed that a cocktail of nine components could reprogram fibroblasts into NSCs (Zhang et al., 2016).

With these considerations, it is crucial to understand the molecular mechanisms behind how autophagy contributes to reprogramming technology and evaluating the benefits of modulating autophagic pathways in reprogramming.

## Conclusion

Autophagy has historically been considered a cellular housekeeping process to maintain cellular homeostasis under various conditions such as nutrient starvation. In this case, autophagy supplies an alternative source of energy for cells. Our knowledge about autophagy-mediated activities has evolved over the years. Several lines of evidence declare that autophagy regulation is strongly associated with the metabolic state of cells. In the case of stem cells, autophagy plays a crucial role in maintaining stem cell homeostasis, function, survival of the long-lived population of stem cells. Additionally, autophagy has the potential to affect cell fate decisions by influencing mitochondrial dynamics, energy production, and epigenetic modulation.

Indeed, autophagy can be a protective mechanism for stem cells from cellular stress due to the reduced potential of stem cell regeneration and increased degenerative diseases in aging. This further proposes that autophagy may be a potential target in regenerative medicine. However, little is known about the contribution of autophagy in HSCT. Autophagy is involved in responses to physiological stress in various ways, depending on the cell type and stimulus. HSCT-induced effects can be influenced by autophagy regulation. In this context, multiple events are more susceptible as follows: including the stress of pre-transplant conditioning, reconstitution of hematopoiesis following transplantation, cytokine-induced responses, antigen processing/presentation, and subsequent differentiation and survival. According to most research, promoting autophagy after HSCT may benefit transplant outcomes.

Given that GVHD has complicated pathology, autophagy plays a crucial role in limiting inflammation and promoting survival in this regard. On the contrary, autophagy may act conversely and lead to cell death. Accordingly, future research is needed to develop pharmacological interventions for modulating specific autophagic networks and cellular targets in conjugation with supportive care in order to improve HSCT outcomes.

## Abbreviations

Atg, autophagy-related genes; AMPK, adenosine monophosphate-activated protein kinase; APCs, antigen-presenting cells; BMT, bone marrow transplantation; CsA, cyclosporine A; DEGS1, delta 4-desaturase, Sphingolipid 1; ER, endoplasmic reticulum; GVHD, graft-versus-host disease; GVL, graft-versus-leukemia; G-CSF, granulocyte colony-stimulating factor; HSCT, hematopoietic stem cell transplantation; HSCs, hematopoietic stem cells; HDACis, histone deacetylase inhibitors; iPSCs, induced pluripotent stem cells; LC3-PE, light chain 3-phosphatidyl ethanolamine; mTOR, mammalian target of rapamycin; PI3KC3-C1, class III phosphoinositide 3-kinase complex I; PAS, pre-autophagosomal structure; ROS, reactive oxygen species; SMER28, small-molecule enhancer of rapamycin 28; Tregs, regulatory T cells; Th1, T-helper type 1; UPS, ubiquitin-proteasome system; Vps, vacuolar protein sorting.
